# Genetic polymorphism of the *OPG* gene associated with breast cancer

**DOI:** 10.1186/1471-2407-13-40

**Published:** 2013-01-31

**Authors:** Jasmin Teresa Ney, Ingolf Juhasz-Boess, Frank Gruenhage, Stefan Graeber, Rainer Maria Bohle, Michael Pfreundschuh, Erich Franz Solomayer, Gunter Assmann

**Affiliations:** 1 Gynecology, Obstetrics and Reproductive Medicine, University Medical School of Saarland, 66421, Homburg/Saar, Saarland, Germany; 2 Internal Medicine II, University Medical School of Saarland, 66421, Homburg/Saar, Saarland, Germany; 3 Institute of Medical Biometry, Epidemiology and Medical Informatics, Saarland University, 66421, Homburg/Saar, Saarland, Germany; 4 General and Surgical Pathology, University Medical School of Saarland, 66421, Homburg/Saar, Saarland, Germany; 5 Internal Medicine I, José-Carreras-Center for Immuno- and Gene Therapy, University Medical School of Saarland, 66421, Homburg/Saar, Saarland, Germany; 6 Universitätsklinikum des Saarlandes, Klinik für Frauenheilkunde, Geburtshilfe und Reproduktionsmedizin, Kirrbergerstr, 66421, Homburg/Saar, Germany

**Keywords:** Breast cancer, Case control study, OPG, Polymorphism, RANK, RANKL, rs3102735

## Abstract

**Background:**

The receptor activator of NF-κB (RANK), its ligand (RANKL) and osteoprotegerin (OPG) have been reported to play a role in the pathophysiological bone turnover and in the pathogenesis of breast cancer. Based on this we investigated the role of single nucleotide polymorphisms (SNPs) within *RANK*, *RANKL* and *OPG* and their possible association to breast cancer risk.

**Methods:**

Genomic DNA was obtained from Caucasian participants consisting of 307 female breast cancer patients and 396 gender-matched healthy controls. We studied seven SNPs in the genes of *OPG* (rs3102735, rs2073618), *RANK* (rs1805034, rs35211496) and *RANKL* (rs9533156, rs2277438, rs1054016) using TaqMan genotyping assays. Statistical analyses were performed using the χ^2^-tests for 2 x 2 and 2 x 3 tables.

**Results:**

The allelic frequencies (OR: 1.508 CI: 1.127-2.018, p=0.006) and the genotype distribution (p=0.019) of the *OPG* SNP rs3102735 differed significantly between breast cancer patients and healthy controls. The minor allele C and the corresponding homo- and heterozygous genotypes are more common in breast cancer patients (minor allele C: 18.4% vs. 13.0%; genotype CC: 3.3% vs. 1.3%; genotype CT: 30.3% vs. 23.5%). No significantly changed risk was detected in the other investigated SNPs. Additional analysis showed significant differences when comparing patients with invasive vs. non-invasive tumors (*OPG* rs2073618) as well as in terms of tumor localization (*RANK* rs35211496) and body mass index (*RANKL* rs9533156 and rs1054016).

**Conclusions:**

This is the first study reporting a significant association of the SNP rs3102735 (*OPG*) with the susceptibility to develop breast cancer in the Caucasian population.

## Background

Breast cancer is one of the most common malignancies in women, leading to distant metastases in patients with advanced disease, particularly in liver, lung and bone. Bone metastases are associated with hypercalcemia, pathologic fracture, spinal cord compression, pain and reduced quality of life [1]. The discovery of receptor activator of NF-κB (RANK), its ligand RANKL and osteoprotegerin (OPG) has contributed significantly to the understanding of the physiological bone turnover. A functional interaction between RANKL, a member of the tumor necrosis factor (TNF) ligand superfamily and RANK, its cognate TNF-receptor is essential for osteoclast differentiation, survival and activation [2].

RANKL, a type II homotrimeric transmembrane protein, is expressed by osteoblasts, osteocytes, bone marrow stromal cells, Tcells and various tumor cells, e. g. myeloma and breast cancer [3-6]. The type-I homotrimeric transmembrane protein RANK is not only expressed by osteoclast, Tcells, dendritic cells, endothelial cells, and mammary glands but also by cancer cells including prostate and breast [7-11]. RANKL- or RANK-deficient mice develop osteopetrosis resulting from a lack of osteoclasts and absence of bone resorption [12,13]. OPG is a secreted homodimeric glycoprotein from the TNF receptor family, lacking a transmembrane domain and has homology to the CD40 protein [14]. OPG neutralizes RANKL, which leads to a reduced RANK-RANKL interaction, thus inhibiting osteoclastogenesis [6,15]. Transgenic mice overexpressing OPG show increased bone mass (osteopetrosis) as a result of reduced osteoclasts [14], whereas OPG-deficient mice are characterized by massive osteoclast activity and osteoporosis [16]. With regard to tumor development, OPG is discussed to be a positive regulator of microvessel formation and to promote neovascularisation [17] and might therefore have an influence on tumor progression. Moreover OPG overexpression by breast cancer cells increased cell proliferation and tumor growth *in vivo*[18].

A disturbed RANKL/OPG ratio was found in a spectrum of skeletal diseases (e. g. rheumatoid arthritis, osteoporosis, bone metastases) characterized by extensive osteoclast activity. Additionally, the RANK/RANKL pathway has intrinsic functionality in mammary epithelium development. Mice that are deficient for RANK or RANKL did not develop lactating mammary gland [8]. Recently, two groups have found that RANKL has not only a fundamental role in the normal physiology of the mammary gland, but may also be crucial for breast cancer development [19,20]. These data support earlier results, where RANKL was shown to play a role in breast cancer cell migration into bone [21] and underscore the potential use of RANKL inhibition in the prevention of breast cancer development. Based on its pivotal role in the bone remodeling process, RANKL has become a therapeutic target. A monoclonal antibody against RANKL, denosumab, has been approved for the treatment of postmenopausal osteoporosis and bone metastasis in breast cancer [22,23].

In summary, the functional properties of the RANK/RANKL/OPG pathway suggest an important effect of the genes on the pathogenesis of breast cancer. These findings led us to investigate the link between seven single nucleotide polymorphisms (SNPs) in the genes of *RANK*, *RANKL* and *OPG*, all possibly associated with functional alterations, and breast cancer risk.

## Methods

### Study populations

A total of 703 participants consisting of 307 female breast cancer patients and 396 gender-matched healthy controls were enrolled in this study (Table 1). All patients and controls were of central European Caucasian ethnicity. Breast cancer patients were collected from the Department of Gynecology, Obstetrics and Reproductive Medicine of Saarland University Medical School, Homburg/Saar, Germany. Controls were either recruited from the Departments of Gynecology, Obstetrics and Reproductive Medicine (n=47), Internal Medicine II (n=163) or the Institute for Transfusion Medicine (n=186) of Saarland University Medical School, Homburg/Saar, Germany. The local ethics committee of the Medical Association from the Saarland (reference number: 162/11) approved the study and all individuals in the study gave written informed consent. The study was carried out in compliance with the Helsinki Declaration.

**Table 1:** Characteristics of study population

**Clinical parameters**	**Breast cancer patients (n=307)**	**Healthy controls (n=396)**
Age (median) in years ^k^	56 (22-91)	45 (18-88)
Menopausal status	n=287	
*Premenopausal*	88 (31%)	
*Postmenopausal*	179 (62%)	
*Perimenopausal*	20 (7%)	
*Unknown*	20	
Tumor growth	n=303	
*Invasive*	275 (91%)	
*Non-invasive*	28 (9%)	
*Unknown*	4	
Localization	n=306	
*Right*	123 (40%)	
*Left*	173 (57%)	
*Bilateral*	10 (3%)	
*Unknown*	1	
Type ^a, b^	n=255	
*Ductal*	189 (74%)	
*Lobular*	34 (13%)	
*Other types*	32 (13%)	
*Unknown*	21	
Tumor size (T) ^a, b, c^	n=229	
*T1 (< 2 cm)*	142 (62%)	
*T2 (>/= 2 cm – 5 cm)*	76 (33%)	
*T3 (</= 5 cm)*	6 (3%)	
*T4 (infiltration of the chest*	5 (2%)	
*wall/skin)*		
*Unknown*	24	
Nodal status (N) ^b, c^	n=250	
*N+*	75 (30%)	
*N-*	175 (70%)	
*Unknown*	36	
Distant metastases (M)	n=292	
*M+*	16 (5%)	
*osseous*	10 (3%)	
*M-*	276 (95%)	
*Unknown*	15	
Tumor grading (G)	n=245	
*G1*	16 (6%)	
*G2*	161 (63%)	
*G3*	78 (31%)	
*Unknown*	49	
Estrogen receptor (ER) ^d^	n=275	
*ER+*	224 (81%)	
*ER-*	51 (19%)	
*Unknown*	32	
Progesterone receptor (PR) ^b, d^	n=274	
*PR+*	193 (70%)	
*PR-*	81 (30%)	
*Unknown*	32	
Her-2 ^a, b, e^	n=208	
*Her2+*	42 (20%)	
*Her2-*	166 (80%)	
*Unknown*	67	
Ki67 ^a, b, f^	n=187	
*Ki67+*	84 (45%)	
*Ki67-*	103 (55%)	
*Unknown*	88	
CEA ^f^	n=107	
*CEA+*	26 (24%)	
*CEA-*	81 (76%)	
*Unknown*	200	
CA15-3 ^h^	n=215	
*CA15-3+*	81 (38%)	
*CA15-3-*	134 (62%)	
*Unknown*	92	
Body mass index (BMI) ^m^	n=219	
*BMI < 28*	150 (68%)	
*BMI >/= 28*	69 (32%)	
*Unknown*	88	
Subgroup ^a, i^	n=249	
*Triple negative*	22 (9%)	
*Non triple negative*	227 (91%)	
*Unknown*	30	
Subgroup ^a, j^	n=262	
*Risk group*	18 (7%)	
*Non risk group*	244 (93%)	
*Unknown*	15	

^a^Only invasive tumors are included; ^b^Bilateral tumors are only included if both sides had the same result; ^c^Exclusion of cases with neoadjuvant chemotherapy; ^d^Immunoreactive score: 0: negative, 1-12: positive; ^e^Her2 = human epidermal growth factor receptor 2; immunoreactive score 0-2 (FISH negative): negative, 2 (FISH positive)-3: positive; ^f^Ki67 = marker for proliferation (< 13%: negative, >/= 13%: positive); ^g^CEA = carcinoembryonic antigen (tumor marker, < 3 ng/ml: negative, >/= 3 ng/ml: positive); ^h^CA15-3 = tumor marker (< 21 U/ml: negative, >/= 21 U/ml: positive); ^i^Triple negative = ER, PR and Her2 negative; ^j^Risk group: T >/= 2, G3, ER negative; FISH = fluorescence in situ hybridization; ^k^significant difference (p< 0.001), age-adjusted statistical analysis performed; ^m^BMI >/= 28 was defined as overweight in order to age-adjustment [https://www.uni-hohenheim.de/wwwin140/info/interaktives/bmi.htm].

Case patients were diagnosed as unambiguously having breast cancer through standard clinical and histological findings. Specific cancer characteristics such as histological subtypes, grading, metastasis were not used as a criterion for the inclusion or exclusion of samples.

### SNP selection

The three genes of interest together span more than 120 kb pairs and show only weak to moderate linkage-disequilibrium patterns according to the HapMap data. We have preferentially selected SNPs which might be functionally relevant, either by their location within a potentially regulatory region (3’ untranslated or promoter region, intron-exon boundary) or by altering the amino acid sequence (missense mutation). A total of seven SNPs were analyzed, two within the *OPG* (rs3102735, rs2073618) and *RANK* (rs1805034, rs35211496) gene, respectively, and three within the *RANKL* gene (rs9533156, rs2277438, rs1054016). Table 2 summarizes the chromosomal position and function of the selected SNPs.

**Table 2:** Selected SNPs for genotyping

**Gene**	**SNP number**	**SNP position**	**Allele [major/minor]**	**Function**
*OPG*	rs3102735	chr8: 119965070	T/C	Transition substitution (5’ near region)
*OPG*	rs2073618	chr8: 119964052	G/C	Missense (p.K3N)
*RANK*	rs1805034	chr18: 60027241	T/C	Missense (p.V192A)
*RANK*	rs35211496	chr18: 60021761	C/T	Missense (p.H141Y)
*RANKL*	rs9533156	chr13: 43147671	T/C	Transition substitution (5’ near region)
*RANKL*	rs2277438	chr13: 43155168	A/G	Transition substitution (intron1/exon2 boundary)
*RANKL*	rs1054016	chr13: 43182002	G/T	Transversion substitution (3’ UTR)

RANK = receptor activator of nuclear factor-κB; RANKL = RANK ligand; SNP = single nucleotide polymorphism; OPG = osteoprotegerin.

### Genomic DNA extraction and Genotyping

Genomic DNA was isolated from peripheral blood lymphocytes using QIAamp DNA Blood Mini Kit according to the manufacturer’s protocols (Qiagen, Hilden, Germany). DNA quantity was assessed spectrophotometrically with the Nanodrop ND 1000 (Peqlab, Erlangen, Germany). All SNPs were genotyped using commercial TaqMan assays (assay IDs: rs3102735: C_1971046_10; rs2073618: C_1971047_1; rs1805034: C_8685532_20; rs35211496: C_25473190_10; rs9533156: C_30009803_10; rs2277438: C_25473654_10; rs1054016: C_7444426_10) with TaqMan Genotyping Master Mix on a 7500 real-time PCR cycler (Life Technologies, Darmstadt, Germany) by following the manufacturer’s instructions.

### Statistical analyses

Hardy-Weinberg equilibrium was assessed in each cohort by comparing the observed genotype distribution with the expected one using a χ^2^-test (Institute of Human Genetic, Munich, Germany: http://www.ihg.gsf.de/). The difference in allele and genotype frequencies between patients and healthy controls (as well as between different subgroups) were analyzed using χ^2^-tests for 2 x 2 and 2 x 3 tables, respectively, with Fisher’s exact test. Differences in allele frequencies were quantified by odds ratios (OR) and 95% confidence intervals (CI). With regard to significantly elder breast cancer patients than healthy controls age-adjusted covariate analysis was performed. All p-values are two-sided and p-values <0.05 were considered as statistically significant. All statistical analyses were performed using the SPSS statistical software. Finally, a power analysis was performed using the G power 3.1.3 software. To the best of our knowledge no adjustment for multiple testing was made because analyses were considered exploratory and needing confirmation by an independent set of data. Previous studies have demonstrated that the analyzed SNPs only show a weak to moderate linkage-disequilibrium patterns according to the HapMap data.

## Results

### Subject characteristics

The mean age was 56 years (range 22-91) for the breast cancer patients and 45 (range 18-88) for the healthy controls showing significant difference. Clinical data (e. g. menopausal status, body mass index (BMI)) and specific cancer characteristics such as localization, histological subtypes, tumor size, metastasis, grading, proliferation index as well as hormone receptor and Her2 expression are listed in Table 1. The tumor markers carcinoembryonic antigen (CEA) and CA15-3 were measured routinely in the blood of preoperative patients. Invasive ductal carcinomas (74%) with a size smaller 2 cm (T1, 62%) and without metastases (nodal negative: 70%, no distant metastases: 95%) at first diagnosis were most frequently. Additionally, most tumors expressed estrogen (81%) and progesterone receptors (70%), as expected, while Her2 was negative in most cases (80%) (Table 1).

### Allele and genotype frequencies and risk of breast cancer

The genotype distributions for all seven SNPs were in the Hardy-Weinberg equilibrium. Table 3 summarizes the results of all SNP analyses in the genes encoding for *OPG* (rs3102735, rs2073618), *RANK* (rs1805034, rs35211496) and *RANKL* (rs9533156, rs2277438, rs1054016). Allelic and genotype frequencies in breast cancer patients were compared to healthy controls.

**Table 3:** **Association of allele and genotype frequencies of *OPG*, *RANK *and *RANKL *in patients with breast cancer and healthy controls**

**SNP**	**Alleles / Genotypes**	**Breast cancer**	**Healthy controls**	**OR (95**% **CI)**	p-value*
*OPG* rs3102735		n=614 (%)	n=784 (%)		
Alleles	C	113 (18.4%)	102 (13.0%)	1.508	**0.006**
	T	501 (81.6%)	682 (87.0%)	(1.127-2.018)	
		n=307 (%)	n=392 (%)		
Genotypes	CC	10 (3.3%)	5 (1.3%)		**0.019**
	CT	93 (30.3%)	92 (23.5%)		
	TT	204 (66.4%)	295 (75.3%)		
*OPG* rs2073618		n=614 (%)	n=786 (%)		
Alleles	C	269 (43.8%)	357 (45.4%)	0.937	0.552
	G	345 (56.2%)	429 (54.6%)	(0.758-1.159)	
		n=307 (%)	n=393 (%)		
Genotypes	CC	57 (18.6%)	77 (19.6%)		0.810
	CG	155 (50.5%)	203 (51.7%)		
	GG	95 (30.9%)	113 (29.7%)		
*RANK* rs1805034		n=614 (%)	n=790 (%)		
Alleles	C	291 (47.4%)	362 (45.8%)	1.065	0.590
	T	323 (52.6%)	428 (54.2%)	(0.862-1.316)	
		n=307 (%)	n=395 (%)		
Genotypes	CC	73 (23.8%)	78 (19.7%)		0.334
	CT	145 (47.2%)	206 (52.2%)		
	TT	89 (29.0%)	111 (28.1%)		
*RANK* rs35211496		n=614 (%)	n=792 (%)		
Alleles	T	122 (19.9%)	141 (17.8%)	1.145	0.335
	C	492 (80.1%)	651 (82.2%)	(0.875-1.499)	
		n=307 (%)	n=396 (%)		
Genotypes	TT	12 (3.9%)	9 (2.3%)		0.423
	TC	98 (31.9%)	123 (31.1%)		
	CC	197 (64.2%)	264 (66.7%)		
*RANKL* rs9533156		n=614 (%)	n=788 (%)		
Alleles	C	280 (45.6%)	369 (46.8%)	0.952	0.666
	T	334 (54.4%)	419 (53.2%)	(0.770-1.176)	
		n=307 (%)	n=394 (%)		
Genotypes	CC	68 (22.1%)	82 (20.8%)		0.387
	CT	144 (46.9%)	205 (52.0%)		
	TT	95 (30.9%)	107 (27.2%)		
*RANKL* rs2277438		n=614 (%)	n=788 (%)		
Alleles	G	109 (17.8%)	132 (16.8%)	1.073	0.669
	A	505 (82.2%)	656 (83.2%)	(0.812-1.418)	
		n=307 (%)	n=394 (%)		
Genotypes	GG	8 (2.6%)	9 (2.3%)		0.866
	GA	93 (30.3%)	114 (28.9%)		
	AA	206 (67.1%)	271 (68.8%)		
*RANKL* rs1054016		n=614 (%)	n=786 (%)		
Alleles	T	258 (42.0%)	345 (43.9%)	0.927	0.514
	G	356 (58.0%)	441 (56.1%)	(0.749-1.147)	
		n=307 (%)	n=393 (%)		
Genotypes	TT	57 (18.6%)	73 (18.6%)		0.543
	TG	144 (46.9%)	199 (50.6%)		
	GG	106 (34.5%)	121 (30.8%)		

CI = confidence intervals; RANK = receptor activator of nuclear factor-κB; RANKL = RANK ligand; OPG = osteoprotegerin; OR = odds ratio; *χ2-tests for 2x2 tables (alleles) and for 2x3 tables (genotypes), respectively.

The allelic frequencies (OR: 1.508 CI: 1.127-2.018, p=0.006) as well as the genotype distribution (p=0.019) of the *OPG* SNP rs3102735 differed significantly between breast cancer patients and healthy controls. The minor allele C was more frequent in breast cancer patients (18.4%) compared to the control group (13.0%). In addition, the homozygous genotype CC of the minor allele as well as the heterozygous genotype CT were more frequent in the breast cancer group (3.3% and 30.3%) compared to the controls (1.3% and 23.5%) (Table 3). The power analysis revealed a power of 0.79 for the allele frequency and 0.72 for the genotype distribution to detect dependencies (α = 0.05) (Additional file 1: Figure S1). Further statistical analysis revealed that the heterozygous genotype CT as well as the homozygous genotype CC together with the heterozygous genotype CT versus the homozygous genotype TT of the major allele significantly differed between breast cancer patients and controls (CT vs. TT: OR: 1.462, CI 1.042-2.052, p=0.030; [CC + CT] vs. TT: OR: 1.536, CI 1.104-2.135, p=0.011). Due to significantly differences in the median age between controls and breast cancer patients (Table 1) we confirmed these data with a logistic regression using age as a covariate (p=0.005).

No significant differences in the allele frequencies and genotype distributions were found, when the breast cancer patients were compared with the controls for the other SNPs analyzed in this study.

### Association between SNPs within different breast cancer subgroups

Next we examined the association between the distribution of genotypes and allelic frequencies of all analyzed SNPs and clinicopathological data including tumor localization, histological subtypes, tumor size, metastasis, grading, proliferation index, hormone receptor expression, Her2 expression, tumor marker level, menopausal status as well as body mass index at the time of diagnosis (Table 1).

Regarding the two *OPG* SNPs the most interesting result was the significant difference in genotype distribution and allelic frequency of *OPG* rs2073618 between invasive versus non invasive tumors. The homozygous major genotype GG (31.3% vs. 21.4%, p=0.006) as well as the major allele G (57.5% vs. 39.3%, OR 2.088 CI 1.189-3.663, p=0.011) were more frequent in patients with invasive tumors in contrast to non-invasive ones (Table 4).

**Table 4:** Association of allele and genotype frequencies within selected breast cancer subgroups

**SNP**	**Alleles**		**Genotypes**		
***OPG *rs2073618**	**G**	**C**	**GG**	**CG**	**CC**
Invasive tumors	316 (57.5%)	234 (42.5%)	86 (31.3%)	144 (52.4%)	45 (16.4%)
Non-invasive tumors	22 (39.3%)	34 (60.7%)	6 (21.4%)	10 (35.7%)	12 (42.9%)
OR (95%CI) p-value*	2.088 (1.189-3.663) p=**0.011**		p=**0.006**		
***RANK *rs35211496**	**T**	**C**	**TT**	**TC**	**CC**
right breast^a^	62 (25.2%)	184 (74.8%)	9 (7.3%)	44 (35.8%)	70 (56.9)
left breast^a^	53 (15.3%)	293 (84.7%)	3 (1.7%)	47 (27.2%)	123 (71.1%)
OR (95%CI) p-value*	1.863 (1.236-2.808) p=**0.003**		p=**0.009**		
***RANKL *rs9533156**	**C**	**T**	**CC**	**CT**	**TT**
BMI >/=28	70 (50.7%)	68 (49.3%)	22 (31.9%)	26 (37.7%)	21 (30.4%)
BMI <28	120 (40%)	180 (60%)	24 (16.0%)	72 (48.0%)	54 (36.0%)
OR (95%CI) p-value*	1.543 (1.029-2.315) p=**0.038**		p=**0.032**		
***RANKL *rs1054016**	**T**	**G**	**TT**	**TG**	**GG**
BMI >/=28	66 (47.8%)	72 (52.2%)	20 (29.0%)	26 (37.7%)	23 (33.3%)
BMI <28	108 (36.0%)	192 (64.0%)	19 (12.7%)	70 (46.7%)	61 (40.7%)
OR (95%CI) p-value*	1.630 (1.083-2.453) p=**0.021**		p=**0.018**		

BMI = body mass index; CI = confidence intervals; RANK = receptor activator of nuclear factor-κB; RANKL = RANK ligand; OPG = osteoprotegerin; OR = odds ratio; *χ2-tests for 2x2 (alleles) and 2x3 (genotypes) tables, respectively; ^a^Exclusion of cases with bilateral tumor involvement.

Data not shown concerning the remaining SNPs stratified into further subgroups according to Table 1.

Another important difference was found with respect to the genotype distribution as well as the allelic frequency comparing the tumor localization (right breast vs. left breast) for the *RANK* SNP rs35211496. The homozygous minor allele T (25.2% vs. 15.3% OR 1.863 CI 1.236-2.808, p=0.003) and the minor allele genotype TT (7.3% vs. 1.7%, p=0.009) were more frequent in patients with tumor involvement of the right breast in contrast to the left side (Table 4).

The allelic frequencies (rs9533156: OR 1.543 CI 1.029-2.315, p=0.038; rs1054016: OR 1.630 CI 1.083-2.453, p=0.021) as well as the genotype distribution (rs9533156: p=0.032; rs1054016: p=0.018) of the *RANKL* SNPs rs9533156 and rs1054016 differed significantly between patients with a higher BMI (>/= 28) compared to patients with a lower BMI (< 28) at the first diagnosis. The minor allele C for SNP rs9533156 and T concerning the SNP rs1054016 were more frequent in patients with a BMI >/= 28 (rs9533156: 50.7%; rs1054016: 47.8%) compared to patients with a lower BMI (rs9533156: 40%, rs1054016: 36%; Table 4).

No significant differences in the allele frequencies and genotype distributions were found in the different subgroup analyses (including distant metastases) for the remaining analyzed SNPs (data not shown).

## Discussion

To the best of our knowledge, this is the first study showing a significant association between the SNP rs3102735 of the *OPG* gene and the susceptibility of breast cancer in Caucasian populations. For the SNP rs3102735 containing the minor allele C as well as for the homo- and heterozygous genotype with the minor allele C, we observed a 1.5-fold increased risk of breast cancer. All other SNPs were not associated with an increased risk for breast cancer. These results suggest a role for the *OPG* gene polymorphism in relation to breast cancer development.

Previous studies showed that genetic variants in the *OPG* locus have been associated with differences in bone mineral density (BMD; [24-33], osteoporotic fractures [28,34], bone turnover [31], bisphosphonate-induced osteonecrosis of the jaw [35], calcaneal quantitative ultrasound (velocity of sound) [36], ankylosing spondylitis development [37] and diabetic charcot neuroarthropathy [38].

In detail, concerning the rs3102735 SNP the G allele was more common among fracture patients [28,34] and patients with lower BMD at the distal radius [30]. Furthermore, there is an association within a subgroup of postmenopausal patients carrying the minor allele and a lower calcaneal velocity of sound [36]. In an earlier study the variation (rs3102735) within the *OPG* gene showed a trend with higher frequency of the minor allele (p=0.076) and responding genotypes (p=0.097) in patients with psoriasis compared to controls without reaching significance [39].

Recently, several genome wide association studies or studies of specific candidate SNPs revealed additional loci to be associated with breast cancer including the same chromosomal region 8q24 as for the *OPG* gene [40-49]. The majority of the association on chromosome 8q24 lies at approximately 128 Mb and is related to several tumor entities (prostate [50], colon [51]) in addition to breast cancer. Each locus within the 128 Mb bears epigenetic enhancer elements and forms chromatin loops with the *myc* proto-oncogene located several hundred kilobases telomeric [52]. A recent meta-analysis revealed an additional locus around 120 Mb on chromosome 8 associated with cancer development [53]. This region is close to the locus of *OPG* rs3102735 SNP (chromosome 8q24 119.965.070), which is associated with breast cancer in our study.

In this context we found a second genetic variation within the rs2073618 SNP of the *OPG* gene when stratifying our breast cancer patients into the subgroups of invasive or non-invasive tumors. However, the impact of the SNPs rs3102735 (5’ near promoter region) and rs2073618, located in the first exon, which encodes the signal peptide of *OPG*, are still unclear. Zhao et al. discussed that the change of the third amino acid from lysine (basic amino acid) to asparagine (uncharged polar amino acid) may have an influence of the OPG secretion from the cells. In their study they found that patients carrying the CC genotype had lower serum level of OPG [33]. In another study, a mutation in a basic amino acid (arginin) in the signal peptide of angiotensinogen drastically affected the secretory kinetics [54]. However, the exact mechanism that the SNP rs2073618 possibly affects the secretory characteristics of OPG needs to be elucidated by further functional studies. Genetic variation within the promoter region of *OPG* (rs3102735) could have an effect on the *OPG* gene expression and thus an influence on tumor development.

Further subgroup analyses according to clinical parameters showed an association with BMI (<28 or >/=28). In general, increased BMI is associated with the risk of some cancers and might differ between sexes and different ethnic populations such as breast cancer [55]. Combined studies revealed that the increase in breast cancer risk with increasing BMI among postmenopausal women is mostly depending on associated increase in bioavailable estradiol [56]. Here we show that the minor allele as well as the genotype of the minor allele of the *RANKL* SNPs rs9533156 and rs1054016 were strongly associated with a higher BMI (>/= 28) in the breast cancer group. Whether obese patients carrying the minor allele from one of the two *RANKL* SNPs have an additionally a higher risk of developing breast cancer remains open in this study due to the lack of BMI data from the control group.

Moreover, we confirmed an asymmetry of breast carcinoma to the left side (57% vs. 40%, Table 1) in accordance with several other studies, which revealed asymmetries in paired organs including breast [57,58], the lungs [59], kidney [60] and testes [61]. Especially for the unsymmetric incidence of breast cancer in favour of the left side, several possible explanations are discussed, including the sleeping habit [62], handedness [63], the preference for breast feeding [64] or breast size [63]. We found that a genetic variation within the rs35211496 *RANK* SNP could have an influence on the tumor localization. Whether this polymorphism has a direct effect on the unsymmetric incidence or indirectly via the breast size can not be answered from this study.

The subgroup analyses stratified into metastatic disease at initial diagnosis showed no significant differences in genotype or allelic distribution. Only 10 of 292 patients were primarily diagnosed with bone metastases. Further studies focusing on skeletal metastases with respect to genetic background are required.

Other genetic variants at the *RANK* locus and/or functionally related genes, including *RANKL* have been associated with differences in bone mineral density [31], rheumatoid arthritis [65,66], aortic calcification [67], age at menarche [68] or Paget′s disease of bone [69]. There is one recent study which showed a genetic variant near the 5′-end of *RANK* (rs7226991) associated with a breast cancer risk [70].

## Conclusion

Our case-control study points to an association of the *OPG* SNP rs3102735 with an increased risk of developing breast cancer. These results could extend the constellation of possible breast cancer risk and might affect early diagnosis.

Future studies are needed, including confirmation of our observation in an independent validation set, to determine the relationship between *OPG* rs3102735 SNP and breast cancer risk in other ethnic groups. Whether this SNP leads to a functional alteration of OPG expression and consequently to an altered RANKL level remains to be shown.

## Abbreviations

BMD: bone mineral density; BMI: body mass index; CEA: carcinoembryonic antigen; CI: confidence intervals; DF: degree of freedom; ER: estrogen receptor; FISH: fluorescence in situ hybridization; G: tumor grading; Her2: human epidermal growth factor receptor 2; M: distant metastases; N: nodal status; OPG: osteoprotegerin; OR: odds ratio; PR: progesterone receptor; RANK: receptor activator of NF-κB; RANKL: receptor activator of NF-κB ligand; SNP: single nucleotide polymorphism; T: tumor size; TNF: tumor necrosis factor.

## Competing interests

JT Ney holds a consultancy position at Novartis. EF Solomayer holds a consultancy position at Novartis and Amgen and received compensation from Novartis, Amgen and Roche. I Juhasz-Boess, F Gruenhage, S Graeber, RM Bohle, M Pfreundschuh and G Assmann declare that they have no competing interests.

## Authors’ contributions

JTN designed and performed the research, collected the clinical data, analyzed data, performed statistical analyses and wrote the paper. IJB helped to design the research and to provide study material. FG provided study material and analyzed data. SG analyzed data and supervised the statistical analyses. RMB provided pathological data of tumor samples and participated in manuscript revision. MP participated in critical manuscript revision and data interpretation. EFS participated in the design of the study, provided study material and financial support for the study. GA designed the research, analyzed data, provided study material, helped to draft the manuscript and provided financial support for the study. All authors read and approved the final manuscript.

## Pre-publication history

The pre-publication history for this paper can be accessed here:

http://www.biomedcentral.com/1471-2407/13/40/prepub

## Supplementary Material

Additional file 1 **Power analysis of the Χ**^**2**^**-tests for the allele frequency (2 x 2 contingency table, a, degree of freedom (DF) = 1) and the genotype distribution (2 x 3 contingency table, b, DF = 2) concerning the rs3102735 *OPG *SNP. Power was calculated by given effect size w, α (0.05) and total sample size (a: 1398; b: 699).** Click here for file
