# Supplementary material for: Genetic polymorphism of the OPG gene associated with breast cancer
Source: BMC Cancer. 2013 Jan 31;13:40. doi: 10.1186/1471-2407-13-40 (PMC3563620; doi:10.1186/1471-2407-13-40)
Supplement: Additional file 1 — Power analysis of the Χ2-tests for the allele frequency (2 x 2 contingency table, a, degree of freedom (DF) = 1) and the genotype distribution (2 x 3 contingency table, b, DF = 2) concerning the rs3102735 OPG SNP. Power was calculated by given effect size w, α (0.05) and total sample size (a: 1398; b: 699). [file 1471-2407-13-40-S1.pdf]

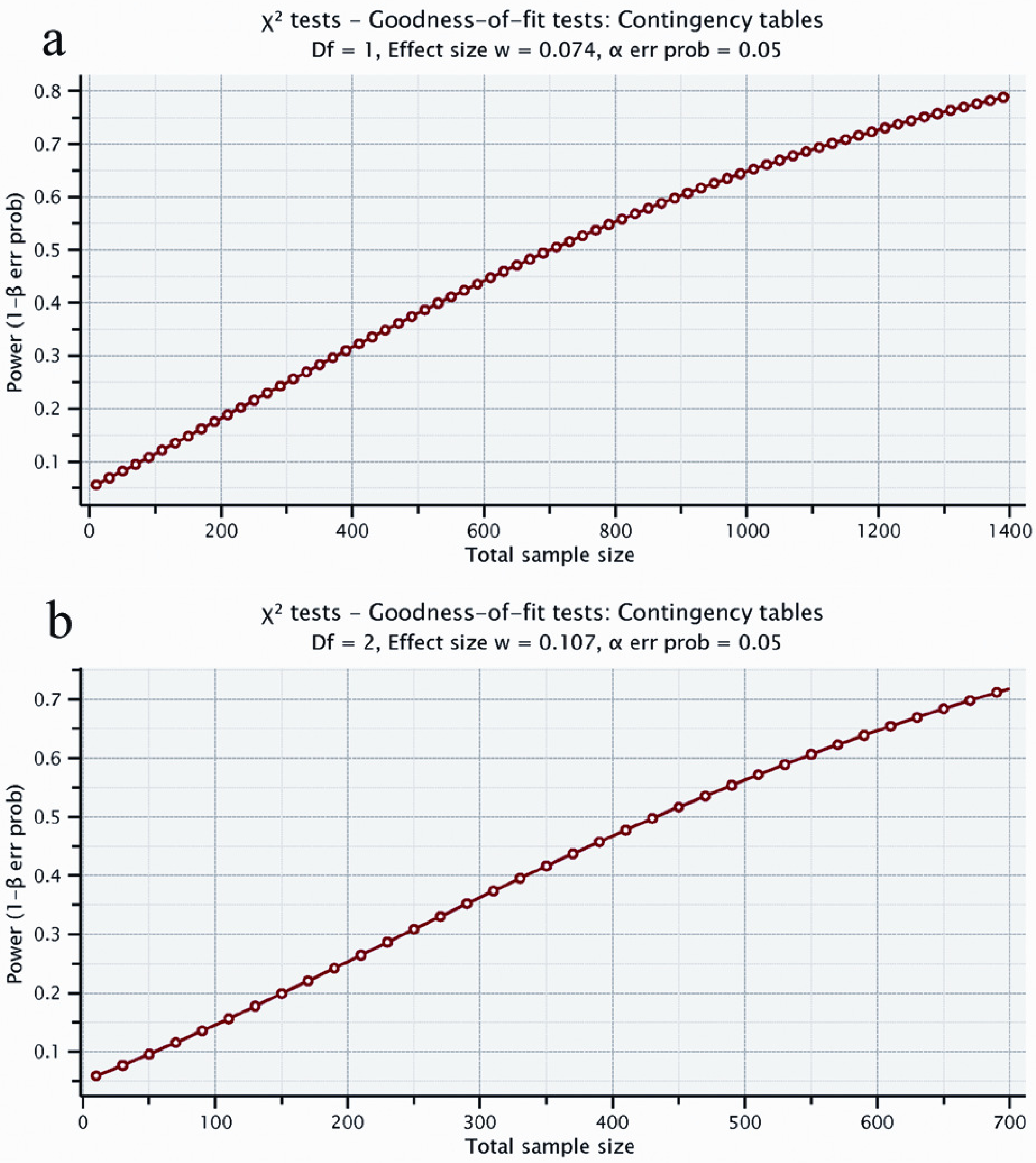
a
χ² tests – Goodness-of-fit tests: Contingency tables
Df = 1, Effect size w = 0.074, α err prob = 0.05
Power (1−β err prob)
0.1
0.2
0.3
0.4
0.5
0.6
0.7
0.8
0
200
400
600
800
1000
1200
1400
Total sample size
b
χ² tests – Goodness-of-fit tests: Contingency tables
Df = 2, Effect size w = 0.107, α err prob = 0.05
Power (1−β err prob)
0.1
0.2
0.3
0.4
0.5
0.6
0.7
0
100
200
300
400
500
600
700
Total sample size
